# Cocreation of Assistive Technologies for Patients With Long COVID: Qualitative Analysis of a Literature Review on the Challenges of Patient Involvement in Health and Nursing Sciences

**DOI:** 10.2196/46297

**Published:** 2023-08-15

**Authors:** Katharina Dalko, Bernhard Kraft, Patrick Jahn, Jan Schildmann, Sebastian Hofstetter

**Affiliations:** 1 Dorothea Erxleben Lernzentrum Faculty of Medicine Martin-Luther-University Halle-Wittenberg Halle (Saale) Germany; 2 Institute for History and Ethics of Medicine, Interdisciplinary Center for Health Sciences Faculty of Medicine Martin-Luther-University Halle-Wittenberg Halle (Saale) Germany; 3 Health Service Research Working Group | Acute Care, Department of Internal Medicine, Faculty of Medicine University Medicine Halle (Saale) Martin-Luther-University Halle-Wittenberg Halle (Saale) Germany

**Keywords:** cocreation, participatory development, transdisciplinary research, technological development, long COVID syndrome, mobile phone

## Abstract

**Background:**

Digital assistive technologies have the potential to address the pressing need for adequate therapy options for patients with long COVID (also known as *post–COVID-19 condition*) by enabling the implementation of individual and independent rehabilitation programs. However, the involvement of the target patient group is necessary to develop digital devices that are closely aligned to the needs of this particular patient group.

**Objective:**

Participatory design approaches, such as cocreation, may be a solution for achieving usability and user acceptance. However, there are currently no set methods for implementing cocreative development processes incorporating patients. This study addresses the following research questions: what are the tasks and challenges associated with the involvement of patient groups? What lessons can be learned regarding the adequate involvement of patients with long COVID?

**Methods:**

First, a literature review based on a 3-stage snowball process was conducted to identify the tasks and challenges emerging in the context of the cocreation of digital assistive devices and services with patient groups. Second, a qualitative analysis was conducted in an attempt to extract relevant findings and criteria from the identified studies. Third, using the method of theory adaptation, this paper presents recommendations for the further development of the existing concepts of cocreation in relation to patients with long COVID.

**Results:**

The challenges of an active involvement of patients in cocreative development in health care include hierarchical barriers and differences in the levels of specific knowledge between professionals and patients. In the case of long COVID, patients themselves are still inexperienced in dealing with their symptoms and are hardly organized into established groups. This amplifies general hurdles and leads to questions of group identity, power structure, and knowledge creation, which are not sufficiently addressed by the current methods of cocreation.

**Conclusions:**

The adaptation of transdisciplinary methods to cocreative development approaches focusing on collaborative and inclusive communication can address the recurring challenges of actively integrating patients with long COVID into development processes.

## Introduction

### Background: Digital Technology as an Answer to Therapy Needs in the COVID-19 Pandemic

In the wake of the COVID-19 pandemic, the rehabilitation of patients in the postacute phase of the disease is an important measure to address the long-term effects. Even though current studies on the so-called *long COVID syndrome* (also known as *post–COVID-19 condition*) provide heterogeneous results and case numbers, they show that a significant number of patients report persistent symptoms after a COVID-19 infection [1,2]. For instance, according to a study by Carfi et al [3] in 2020, approximately 87% of patients who had recovered from COVID-19 reported persistent symptoms, such as fatigue, dyspnea, joint pain, and chest pain, after inpatient treatment. As estimated by the World Health Organization in 2022, in Europe, 20% of patients with COVID-19, even those without the need for inpatient treatment, generally report the aforementioned limitations continuing for at least 3 months after recovery [4]. In addition, psychological symptoms, such as anxiety and stress, appear, and a negative impact on the quality of life caused by the long-term effects can be observed [5,6]. Consequently, a rapid establishment of appropriate therapy and rehabilitation measures is essential. Although the number of people affected remains high even as the pandemic situation continues to ease, there is still insufficient knowledge about long COVID syndrome and a shortage of specialists and therapy programs [6,7].

One of the ways to address the shortage of adequate programs is to develop digital therapy solutions and assistive devices that are applicable in a home setting and can be individually applied without constant supervision by specialist staff. These so-called *digital assistive technologies* in health care refer to digital applications and devices that offer support to people with physical or mental impairments, for example, through motor or sensory functions [8]. In the context of chronic diseases, such as chronic obstructive pulmonary disease, existing studies show positive results for the use of assistive technologies managing respiratory symptoms, fatigue, or anxiety [9,10]. In addition, with regard to long COVID syndrome, digital assistive technologies might include smartphone apps that support the documentation and monitoring of symptoms and internet-based applications that provide instructions for patients in physical rehabilitation programs [11,12]. However, in the context of long COVID, appropriate technologies are still being developed to meet the requirements resulting from the spectrum of symptoms of long COVID syndrome [13,14]. The World Health Organization, for instance, recommends a patient-centered development of rehabilitation measures, digital services, and devices to support the self-care competence of patients [6]. Smits et al [14] argued that digital technologies such as virtual reality offer great potential for the rehabilitation of patients with long COVID. At the same time, however, the rapid development and selection of applications is a major challenge owing to, among other things, a lack of patient-reported information [14]. Given the novelty of the postacute syndrome, the involvement of patients seems even more crucial because researchers, practitioners, and patients are all still learning how to address and manage its symptoms adequately [7]. We argue that well-thought-out structures and methods are needed to meaningfully implement the engagement of vulnerable groups, particularly in the context of the pandemic and unfamiliarity of the disease.

Cocreation provides a framework for the participatory development of technology through an iterative and agile development process primarily involving affected target groups and stakeholders. Cocreative processes, therefore, include the needs and perspectives of not only end users but also all relevant institutions or groups, including patients; universities; companies; public and care institutions; and mediators, such as suppliers. Cocreation, therefore, aims to consider various interests referring to a standard of fairness and the enforcement of democratic development processes in which all relevant stakeholders are transparently integrated [15,16]. This means that, ideally, different stakeholders, especially patients, are actively involved on equal terms in all aspects of the cocreative process, from the idea development phase to the conception of digital solutions to their implementation [17]. In this sense, end users not only play a crucial role in the development process as experts of their own disease but are also recognized as a vulnerable stakeholder group with unique needs and demands [18].

Participatory forms of development, such as cocreation, are already well established in health and nursing sciences. However, an established model for involving patients with long COVID as a vulnerable group in cocreative processes does not currently exist. Available publications have focused on theoretical participatory principles and approaches to research with different stakeholders, as well as the use of creative methods (design thinking and user-centered design) and questions regarding the degree and timing of patient involvement [19-21]. Although some publications evaluate individual participatory projects using a best practice or lessons learned approach in general, a synthesis of the experiences of different projects has been developed in only a few publications so far [22,23]. Furthermore, patients with long COVID are a relatively new patient group, with only a few studies on their involvement in cocreative processes available. Hence, a discussion of guidelines for the integration of patients with long COVID into cocreative research is still lacking. Therefore, this paper explores the tasks and challenges associated with patient involvement in cocreation with the aim of incorporating the findings into the further development of the cocreative approach to technology development in the context of long COVID syndrome. Therefore, we introduce transdisciplinary methods as a solution to overcome the identified challenges.

### Objectives

This study addressed the following research questions with the aim of introducing an adaptation of cocreation for technology development in the context of health and nursing care, with a special focus on patients with long COVID: what are the tasks and challenges associated with the engagement of patients in cocreative design? What are the lessons we can learn in the context of long COVID syndrome?

This main research interest can be divided into the following two subquestions:

1. What are the challenges of involving patients in cocreative approaches to the development of assistive technologies and services in the health care sector?
2. How can cocreative approaches be adapted to address the challenges and barriers identified in the context of long COVID syndrome?

## Methods

### Overview

A literature review following a snowball approach based on the guidelines of Wohlin [24] was conducted to identify publications. Relevant studies were selected by searching 4 different databases. The snowballing approach was chosen as an iterative search strategy to maximize the number of included studies that evaluated the implementation of cocreative approaches focusing on the active involvement of patients. This method was chosen because even though there is a vast number of publications dealing with participatory development, we expected to find only a limited number of publications reporting on the challenges in the implementation process. Therefore, the snowball method was particularly suitable for selecting a relevant portion of the extensive literature on participatory research. This approach also allowed us to identify publications that did not use the search terms applied but still contained information related to the research questions. Finally, the snowball method is an effective way to provide a summary of findings from a specific field, in this case, health and nursing sciences, as cocreative approaches are used in other disciplines as well. Tasks and challenges in involving patients in cocreative development processes were further extracted from the literature using a qualitative content analysis [25].

### Search Strategy

A total of 4 databases, MEDLINE (PubMed), The Cochrane Library, CINAHL, and Google Scholar, were searched with the aim of exploring findings from the field of health and nursing sciences. The search was conducted from March to July 2022. The search terms were identified using the PICo (population, phenomenon of interest, and context) scheme.

The following search terms were used in this process: “participatory development,” “co-creative development,” “participatory design,” “co-creative design,” “co-production,” “patient” (“patients,” “patient group,” “patient groups”), and “patient involvement.”

Google Scholar was manually searched for relevant studies [26].

### Identification of a Start Set

The start set for the snowballing approach was identified by applying the search strategy and the following inclusion and exclusion criteria to the databases listed earlier. English and German publications that dealt with the challenges of cocreative or participatory processes in which patients were actively involved in the development of health care technologies or services were included (Textbox 1). Articles presenting cocreative approaches without elaborating on the implementation process with a focus on patient groups or publications regarding the coproduction of policies or research agendas as well as shared decision-making and inpatient care were excluded. Owing to the limited number of existing papers on the challenges and barriers to patient participation in cocreative development processes in health and nursing sciences, the initial search considered all publication types, including conference papers and research reports, to broaden the results. A screening of 34 full texts resulted in 7 (21%) publications, which were included as the start set of the snowballing process.

**Inclusion and exclusion criteria of the study search.**

**Inclusion criteria**

- Studies from the field of health and nursing care
- Cocreative or participatory studies with an active involvement of patients
- Studies with the aim of developing new digital devices or services
- Studies that reported challenges and barriers to the involvement of patients
- Studies that involved patients with long COVID

**Exclusion criteria**

- Participatory studies with no involvement of patients
- Studies in the inpatient care setting
- Studies that did not report process-related tasks and challenges of patient involvement
- Studies that referred to a shared decision-making process or the development of research agendas
- Studies whose full texts were not available

Although we were able to identify a limited number of studies that involved patients with COVID-19 in participatory processes in the initial database search, these did not report methodological or process-related tasks and issues in the involvement of this group of patients [27]. Therefore, we included publications that generally addressed the challenges and barriers to patient engagement and discussed the results in the context of long COVID syndrome.

### Backward and Forward Searches

**Figure 1:**
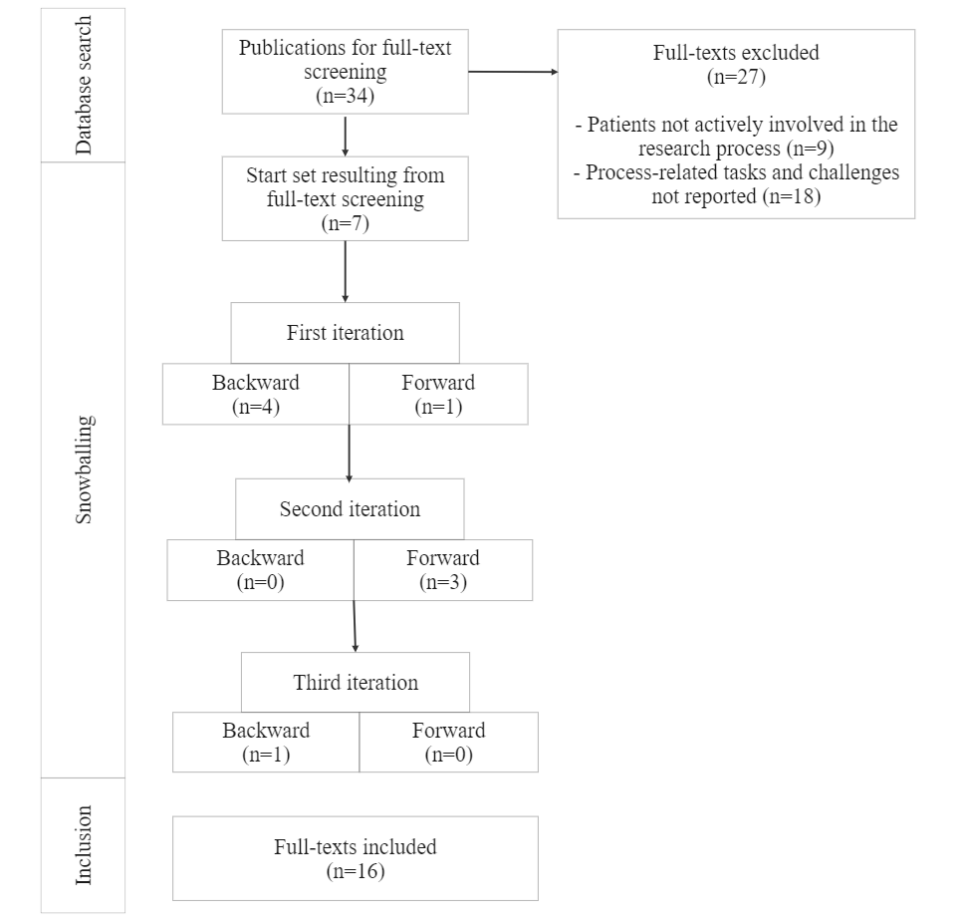
Flowchart of the snowballing process.

After generating the start set, this first set of papers and the papers derived from each following iteration served as a base to identify additional publications until no new relevant papers could be identified (Figure 1). This was achieved through backward and forward snowballing. The main advantage of this approach is the possibility of identifying publications that did not use the search terms applied but still contained information related to the research questions. The backward search refers to the identification of studies from the lists of references of studies already included. For instance, the screening of the reference lists of the start set resulted in the inclusion of 4 additional publications. By contrast, the forward search, is based on the screening of lists of papers citing the papers derived from the initial search (start set) and those derived from the subsequent iterations. Backward and forward searches were performed in 3 iterations. A total of 5 papers were identified via backward snowballing, and 4 papers were identified via forward snowballing. Thus, including the start set (7 studies), we considered 16 papers in the qualitative analysis.

### Qualitative Analysis and Theory Adaptation

A thematic content analysis was performed to extract and summarize the findings from the literature through an inductive process. Two researchers (KD and BK) derived evidence on tasks, challenges, and barriers related to patient involvement in cocreative development processes in health and nursing sciences from the literature included in the analysis. The identified aspects were then categorized thematically using a thematic content analysis approach. Themes were inductively generated from the literature and discussed within the research team (KD and BK) [25].

The results of the literature review are then discussed in the context of initial findings on patients with long COVID. Finally, using the theory adaptation method described by Jaakkola [28] as a methodological guideline, we suggest an adaptation of the cocreation approach to address the identified challenges. The theory adaptation method aims to extend an existing concept by adding methods derived from another theoretical approach to address existing gaps or problems. For this purpose, the initial concept is defined, and problems are identified. A second theoretical concept is then discussed as a proposed solution. In this paper, the concept of cocreative technology development involving patients is contrasted with the theoretical approach of transdisciplinary research.

## Results

### Overview of the Included Publications

The initial database search resulted in 7 publications, which were included in the snowballing process. In the following backward and forward searches, 9 additional papers were identified for inclusion (Figure 1), resulting in a total of 16 publications. These 16 include mainly qualitative studies (n=7, 44%) and 1 (6%) quantitative study as well as lessons learned (n=2, 12%) from several participatory projects. We further identified 3 systematic reviews [22,23,29]. We also included 1 research report and 2 conference papers [30-32]. The years of publication ranged from 2016 to 2022, suggesting that the topic of cocreation and participatory development involving patients in health and nursing care is rather new. Furthermore, studies on this topic are mainly from English-speaking (Canada, Ireland, and the United States) or German-speaking (Germany, Switzerland, and Austria) countries (Table 1).

**Table 1:** Overview of the included publications.

Study, year			Country		Study design		Objective		Participants, n
**Studies derived from the initial search (start set)**									
	Arboleda et al [33], 2021	Germany		Mixed methods		Evaluation of and lessons learned from participatory projects aiming at developing assistive robotic technology for people with physical disabilities		N/A^a^	
	Amann and Sleigh [29], 2021	Switzerland		Systematic review		Systematic review of studies involving vulnerable groups in coproduction with a focus on health care services		N/A	
	Ball et al [30], 2019	The United States		Mixed methods		Outlining the findings on motivations for, approaches to, and challenges in the involvement of patients and the public in health care research from the literature and practice		N/A	
	Israilov and Cho [34], 2017	The United States		Qualitative study		Challenges and benefits of patient-centered cocreation in the health care sector		N/A	
	Kaisler and Missbach [35], 2020	Austria		Qualitative study		Developing a guideline for cocreative research while involving different stakeholders		24	
	Roura [36], 2020	Ireland		Qualitative study		Development of a conceptual framework to outline power inequalities in participatory health research involving patients and the public		N/A	
	van Dijk-de Vries et al [37], 2020	The Netherlands		Participatory action research		Participatory development of a framework for cocreation in the health care sector		21	
**Studies derived from the subsequent iterations**									
	Ashcroft et al [38], 2016	The United Kingdom		Quantitative study		Experiences of patients and carers with participatory research in the health care sector		143	
	Black et al [39], 2018	Canada		Qualitative study		Exploring the experiences of patients and informal caregivers acting as partners in research teams		19	
	Egid et al [40], 2014	Various		Qualitative study		Experiences of participatory health researchers working with nonacademic research partners, such as patients		59	
	Hamilton et al [41], 2017	Canada		Qualitative study		Development of an empirically based conceptual framework for patient engagement in research using the experiences of patients as a guideline		18	
	Lee et al [32], 2017	The United States		Qualitative study		Participatory design of social robots involving older adults with depression and health care staff		15	
	Ostrowski et al [31], 2021	The United States		Qualitative study		Developing guidelines for the codesign of robots and the establishment of long-term relationships with patients as part of collaborative development		28	
	Voorberg et al [23], 2015	The Netherlands		Systematic review		Systematic review of publications concerning cocreation with citizens, that is, patients		N/A	
	Weiss and Spiel [42], 2022	Austria		Qualitative study		Lessons learned from 2 projects involving laypersons in the cocreative development of robots		N/A	
	Zibrowski et al [22], 2021	Canada		Systematic review		Rapid realist review of the literature concerning the mechanisms of developing and maintaining a collaborative relationship between academics and patients as part of patient-centered research in the health care sector		N/A	

^a^N/A: not applicable.

All publications referred to cocreation and participatory development as approaches to involve users in the design process to gain a better-accepted, user-friendly product or service that precisely addresses the needs of the target groups [22,23,30-34,36,37,39-42]. The timing and intensity of patient involvement differed between studies. However, the discourse generally refers to iterative processes in which patient groups are encouraged to contribute their specific views and criticism of ideas and innovations with the aim of further developing and improving services. Kaisler and Missbach [35] even suggested the involvement of different stakeholders, such as patients, before the actual research project begins, that is, during the conceptualization of project goals and applications for funding.

The cocreative approach to designing and creating new products comes with specific challenges. These range from organizational to motivational to ethical challenges. Consequently, these particular barriers and challenges need to be addressed and reflected upon for a successful implementation of the approach. For instance, Coulter [43] identified aspects such as collaboration, communication, information sharing and power structures as crucial to the general engagement of patients in health care–related research as part of a literature review. The categories discovered were used as a guideline for reporting our findings [43].

### Collaboration

In cocreative projects, diverse research teams, among which working practices and interests vary, are involved on the one hand, and nonprofessional participants, such as patients, work together with practitioners, on the other hand. Therefore, understanding others’ perspectives and needs and joint decision-making are general tasks in cocreative processes, where the autonomy and self-determination of the affected groups should be ensured [22,23,30-34,36,37,39-42,44]. As a result, the translation between different perspectives and backgrounds is a key condition for a successful, cocreative, inclusive, and multiprofessional project. Therefore, cocreation is, recognized as an attempt to actively involve and mediate between stakeholders who develop and stakeholders who use technologies and services [22,23,33,34,36,38].

However, a common criticism of cocreative or participatory projects is that patients are merely involved as a tick-box exercise, without their contributions having much influence on the research process [36,38,40]. In addition, patients themselves report a lack of insight into the results or impact of their contributions on the research process, suggesting a rather passive role for patients in the research team [23,39,40]. In line with this, several publications stressed the hierarchical barriers between professionals, academic or medical personnel, and patient groups, which prevent meaningful collaboration [22,23,33,36].

Roura [36] also draws attention to the heterogeneity within patient groups, which represent different interests and needs, even if they are affected by the same clinical syndrome. These differing interests can hinder collaboration among patient participants and the formation of a group identity of patients as a stakeholder. Furthermore, patients criticized that it was not clear to them which role they would have to fulfill and what was expected of them in the research process [39-41]. Patient groups usually lack experience and knowledge regarding and skills for the implementation of research projects and cocreative development compared with professional participants [22,30,39-41]. However, for a successful collaboration within a cocreative project, all participants should be properly informed about the aspired mode of cooperation, its formats, and decision-making processes [36,42]. Therefore, Egid et al [40] emphasized the need to educate patients about what can be expected of the research process to foster active involvement.

### Information Sharing and Communication

The reason for patient involvement is their specific experience with a particular disease and the knowledge they gained when they were affected and treated [30]. This knowledge gained through “lived experience” includes the awareness of the source of the symptoms, certain needs that come along with them, or the effects of the treatment. All of these are important factors to consider to ensure the acceptability and feasibility of innovative assistive technologies [30]. Therefore, the specific knowledge of patients contributes significantly to the outcome of the project and should be respected as such [30,39,40].

Discrepancies among participants regarding subject-specific knowledge, including the use of a particular language, technical terms, and working practices, are one of the main challenges reported in the context of cocreative research [22,32,33,35,36,38,41]. This can lead to barriers for patients to actively engage in development and to feelings of vulnerability [22,33]. Therefore, the creation of a trustworthy and safe environment where contributions by patients are valued and not challenged by experts to enable meaningful exchange was identified as essential [31,39-42]. Moreover, some publications identified mutual learning as one of the main tasks in participatory design projects. Researchers are studying the perspectives and needs of patients, whereas patients need the opportunity to learn about design processes and might not be familiar with the specific innovative technology. Therefore, sufficient time to feel comfortable using and commenting on the respective devices or software is necessary [31-33,40-42]. Roura [36] points out that a lack of access to information can also be a reason for patients to not feel empowered to actively participate in the process. Aspects such as team formation and efforts to get to know each other can be helpful in this regard. However, these are time-consuming tasks that often fall short in closely timed projects [31,42].

Knowledge transfer is also connected with the degree of involvement aimed at by project partners. To what degree is the collaboration with patients beneficial in light of the project goals? How involved do patients want to be? Patient groups need to be informed about all aspects of the research process to be in a position to decide their degree of involvement according to their preferences [31,39,41].

### Power

Established power structures and economic factors, such as project funding, come with certain inequalities. Some partners, mostly researchers and business partners, are involved earlier than other stakeholders. They set up the project, including the selection of topics, initial research questions, and composition of the project members. They probably also establish the funding of the project. Consequently, it is necessary for the leading party to be aware of this fact and try to involve others, especially the patient group, as early as possible in deciding on the common goal. This helps the partners identify with the project. If patient groups are intrinsically motivated, the project becomes a shared responsibility. This also leads to the questions of how power can be shared equally in the decision-making process and how different perspectives and interests can be openly communicated [36,39,40,42]. Hierarchical structures due to social or professional status and differences in the levels of specific knowledge impede the addressal of these questions [22,23,32,36,38,40]. This can also be the case for intragroup dynamics because differences in the degree of involvement and contributions among patients may occur [39].

Moreover, the involvement of patients requires the consideration and discussion of the vulnerability of patient groups. According to the Declaration of Helsinki, a vulnerable group or person “may have an increased likelihood of being wronged or of incurring additional harm” [45]. Nevertheless, one must be careful not to frame the vulnerable person per se as the weak partner in cocreation [36]. From the perspective of vulnerable participants, it is, first of all, crucial to inform them sufficiently about the processes in the project and their scope for action. This is the necessary prerequisite for participation in the sense of informed consent [31,35,39,41]. The fair distribution of benefits and burdens among the participating partners is in line with the protection of the most vulnerable members of the cocreative project. This begins with a decision process driven by scientific and project-related reasons and not purely practical reasons, such as accessibility or manipulation [46]. The aim is to establish a relationship of reciprocity where everybody benefits from working together [31,40]. There is a danger of research processes being shaped solely by the research agenda of scientists and physicians, them being the dominant team members, without patient groups having any major influence [36,38,42]. In addition, highly specific decisions, such as hardware choices, are often not made collaboratively but solely by designers and manufacturers with technical expertise [42]. Ostrowski et al [31] and Egid et al [40] suggested that patients should have the opportunity to reflect on the collaboration within the team and their role, as well as the research process, as part of cocreative approaches. Kaisler and Missbach [35] even called for an independent reference person for patients as “a neutral contact person that can be addressed in case of complaints and concerns.”

## Discussion

### Principal Findings

#### Summary

In light of the COVID-19 pandemic, digital assistive devices, such as phone apps or monitoring devices for the home environment, have become even more relevant, given the lack of access to therapy or rehabilitation measures because of contact restrictions and an overburdening of the health care system [1]. Involving patients with long COVID in the development of new technologies is essential to adequately address their specific needs and conditions [6]. However, our analysis showed that there are various barriers and challenges to consider when involving patients in cocreative development approaches. Therefore, concrete methods have to be established to address these challenges.

#### Involving Patients With Long COVID in Cocreative Projects

The aforementioned challenges in involving patients in the cocreative development are further amplified in the case of patients with long COVID. For example, in light of the diversity of symptoms experienced by as well as the variety of preconditions of patients with long COVID syndrome, the questions of group identity and strategies for the recruitment of participants arise [3,5,47]. Patients are incorporated into research projects and cocreative development, as they offer specific knowledge regarding their needs as future users of innovations. Even though this means that cocreation aims to develop new technologies that are accessible to as many people as possible, as pointed out, for instance, by Roura [36], the heterogeneity of the members of the patient group can also hinder the successful formation of a group identity and collaboration within the cocreative team. Furthermore, given the limited knowledge about long COVID syndrome and its symptoms, joint learning within the research and development process, which is emphasized by various studies, is of particular importance. Compared with other chronic diseases, when it comes to long COVID syndrome, researchers are even more dependent on patients as experts, whereas patients themselves are still learning how to deal with their own disease. At the same time, patients experience anxiety and uncertainty, especially because of the lack of knowledge regarding their condition [47].

A common practice in scientific research that is not aimed at clinical care is to recruit participants from existing bodies such as self-help groups and associations. There are several reasons for this: the patients are already organized into a fixed group, often know each other beforehand, and can be easily addressed [19]. Even though more support groups for patients with long COVID have gradually been established, the majority of these groups exist as web-based groups on Slack (Slack Technologies) or Facebook (Meta Platforms). This was especially true at the beginning of the pandemic and against the backdrop of lockdown restrictions [47]. Davis et al [1], for instance, recruited patients with long COVID for their survey across a variety of support groups, nonprofits, and web-based platforms. They were particularly able to reach social networks that discussed information about long COVID across countries in forums and groups. In addition, the majority of people recruited were from the United States and the United Kingdom, suggesting that it is mainly people from these regions who use the support services. However, European and Asian participants were scarcely represented, even though the respective services were also available in the local language [1]. These initial findings suggest that only a few fixed self-help groups existed at the time, that these were limited to certain regions, and that the majority of information exchange took place in the digital space. This makes the recruitment of patients with long COVID more difficult. However, this also means that different hurdles arise for the target group depending on the method of involvement. Recruiting via digital channels initially means that some groups of people who are not digitally active will probably be excluded from participation [27]. Patients recruited through digital channels or through clinics and doctors’ offices meet for the first time in the context of the research project and may, therefore, find it difficult to form an identity as a group.

It is apparent that there are numerous factors to consider when involving patients with long COVID in a cocreative design process. The management and organization of such a diverse research team, the decision-making process, and the communication of knowledge and perspectives seem to be the key factors in this regard [30,48]. Although cocreative approaches are committed to an inclusive and collaborative operating principle, there are currently no set models for the methodological and structural implementation of inclusive projects involving patients. Aspects such as team structures, team building, and communication have been shown to be central to the success of participatory projects; however, there are currently no established methods for integrating diverse partners, especially patients, into cocreative processes.

#### Adapting Cocreative Approaches Through Transdisciplinary Methods

We propose extending cocreative approaches using methods from transdisciplinary research with a focus on team building and communication to address the challenges listed earlier. Transdisciplinarity is a concept of scientific research that represents an integrative approach to the implementation of collaborative research projects through the cooperation of different disciplines and industries. The approach is based on the assumption that the specialization of disciplines and expert knowledge of partners from practice represent a great added value and must, therefore, be integrated in the best possible way [49,50]. This applies to practice-oriented projects in particular, which require the integration of specialized knowledge from practice as well as the interests and expectations of various stakeholders [49]. Therefore, transdisciplinary research refers to “a research approach that includes multiple scientific disciplines (interdisciplinary) focusing on shared problems and the active involvement of practitioners from outside academia” [51]. However, recent approaches focus not only on the collaboration of scientists and practitioners but also on the involvement of the public [52].

#### Addressing the Challenges of Involving Patients With Long COVID in Cocreative Technology Development

##### Overview

As transdisciplinary approaches pay particular attention to the formation of team structures and communication within interdisciplinary teams, they can address the aforementioned problems of involving patients as laypersons, on the one hand, and the identified issues of involving patients with long COVID, on the other hand. Even if these methods are approaches to cooperation between different disciplines and one, of course, cannot speak of patients as a separate “discipline,” the challenges addressed (building a team, defining structures, and finding a common goal and common language) are also relevant to patient involvement, as shown by our analysis. Transdisciplinary projects provide structural and operational measures of project management with the aim of team building and the creation of a common basic understanding of goals, also referred to as *integration-supporting measures*. A special focus is placed on communication. Transdisciplinary approaches attribute great importance to preliminary preparations, such as planning and team building, for the integration of different perspectives. Furthermore, a constant discourse and continuous exchange of results are expected, particularly for the successful integration of nonacademic partners [49,50].

##### Team Structure and Roles

Owing to the diversity of transdisciplinary projects, models from this field place great emphasis on team building and the elaboration of team structures and roles right at the beginning of the project. This provides orientation within the team and ensures transparency regarding tasks, roles, and decision-making processes. Practical measures are proposed to establish a team and a common work culture. This includes regular work meetings with the whole team as well those with small groups, especially with the members of individual subgroups of the project. Regular reflection on work processes is also integral [49,50].

To enable various participants to organize themselves and emerge as a common interest group within the research team, it is necessary to consider intragroup team building as part of the participatory process [36]. This is particularly relevant given the diversity of the needs and symptoms of patients. Similarly, problems that arise owing to the vulnerability of patients and a possible inequality within the research team can, thus, be addressed [49].

##### Developing a Common Knowledge Base and Goals

One of the goals of team building and preparation measures is the professional and social integration of various stakeholders, which is a basic prerequisite for joint learning and the development of project results. First and foremost, the joint formulation of the research question as well as the goals and contents of the project are the basis for collaboration. Another important aspect is the formation of a team, that is, the structuring of teams and committees, and the conception of a common communication structure. In the next step, it is important to define a common understanding of terms to prevent misunderstandings resulting from different technical languages and background knowledge [49,50]. Accordingly, a common knowledge base and common goals are developed in a collaborative process as part of the second step of the transdisciplinary model [48,49].

##### Communication as a Key Factor

Communication is central to the implementation of transdisciplinary projects. In these projects, communication not only acts a means of group organization but also establishes the mindset necessary for inclusive interactions, mainly the openness to other perspectives and the so-called *communication cultures*. To achieve successful cooperation, the creation of a project-specific communication culture in which all partners can participate at a low threshold is emphasized. Particular attention is also paid to the intersections between individual groups [49].

The involvement of patients in the planning and execution of care is discussed and applied internationally to improve the experience of those affected and the nursing outcomes. In this context, studies show that particularly trusting and personal relationships and communication have an influence on the success of the participation of patient groups. Therefore, Chambers [48] suggested guidelines to ensure inclusive and continuous communication practices to enhance meaningful patient involvement [52]:

Increasing patient involvement (PI) in care decisions and greater partnerships between patients and health-care professionals (HCPs) will help ensure improved patient safety and enhanced patient satisfaction. To facilitate this, guidelines and frameworks can be helpful, whilst acknowledging and respecting the need for flexibility depending on the care context [48].

The strategies and methods applied can include choosing the right wording, creating transparency in processes, and defining the goals of research projects. Stewart et al [53] also cite the importance of openness; inclusion; and, in particular, reflection on and evaluation of the processes of involving stakeholder groups. In this context, the focus is primarily on the understanding of roles, communication of information, and language used [50,53,54].

#### The Adaptation of Cocreative Approaches

**Figure 2:**
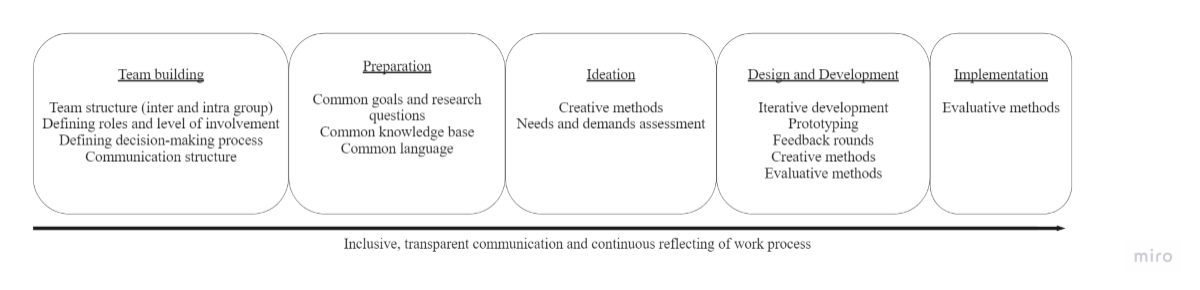
Phases of cocreative research projects complemented by transdisciplinary methods.

The transdisciplinary methods of team building and group preparation can provide a solution to the general challenges of involving patients in cocreation and increased barriers in the context of patients with long COVID. Team building measures can ensure that this so far hardly organized and heterogeneous group of patients with long COVID can develop a team identity for the duration of the research project and, thus, represent their interests. A focus on clear and common communication structures and inclusive communication in the project network can additionally favor the reduction of hierarchies, flow of information, and consideration of discrepancies in specific knowledge. Thus, transdisciplinary methods can complement the early phase of the cocreative research process and provide measures for the formation and preparation of teams. At the same time, emphasis is placed on continuous joint reflection on work processes as well as the integration of all stakeholders through inclusive language and transparency (Figure 2).

### Implications for Research Practices

Various implications arise for research practices from the presented results, particularly for the implementation of research projects in the context of pandemics. Additional team building, participant preparation, and communication measures are necessary to ensure meaningful patient engagement. Sufficient time and resources to implement these must be available for cocreative projects. This is particularly relevant in the context of the patient group with long COVID syndrome, as they can be expected to need additional time to form a group and adapt to their role within the research team. Furthermore, conventional methods of recruitment, such as the involvement of existing support groups, cannot usually be resorted to either; therefore, new strategies of involvement and group formation must be developed.

### Limitations

The limitations of this study include the lack of studies involving patients with long COVID. Consequently, at this time, only findings from studies with other patient groups can be applied to the patient group with long COVID. In addition, the methodological approach to the implementation of cocreative processes and concrete measures for involving patient groups is usually poorly described or not described at all in most existing publications. However, this would be essential for the reflection on and further development of the corresponding processes.

### Conclusions

This study shows that there are certain challenges associated with cocreation as a measure of patient involvement. These range from challenges concerning collaboration and power structures to those concerning group organization. Although participatory study designs can add value, particularly in the context of the pandemic, such as for the development of assistive technologies to support treatment interventions for patients with long COVID, additional hurdles arise for this patient population. These hurdles result particularly from the novelty of the disease and diversity of the spectrum of its symptoms.

Our recommendation for addressing the aforementioned challenges is to adapt the cocreation framework using well-established methods from transdisciplinary research, especially group formation and communication practices. We suggest thus improving the method and process of cocreative technology development. Further research is needed in gathering experience using this adapted method of cocreation, especially with respect to involving patients with long COVID.

## Abbreviations

PICo: population, phenomenon of interest, and context
